# A Combination of Polymethoxyflavones from *Citrus sinensis* and Prenylflavonoids from *Humulus lupulus* Counteracts IL-1β-Induced Differentiated Caco-2 Cells Dysfunction via a Modulation of NF-κB/Nrf2 Activation

**DOI:** 10.3390/antiox12081621

**Published:** 2023-08-16

**Authors:** Ignazio Restivo, Manuela Giovanna Basilicata, Ilenia Concetta Giardina, Alessandro Massaro, Giacomo Pepe, Emanuela Salviati, Camilla Pecoraro, Daniela Carbone, Stella Cascioferro, Barbara Parrino, Patrizia Diana, Carmine Ostacolo, Pietro Campiglia, Alessandro Attanzio, Antonella D’Anneo, Fanny Pojero, Mario Allegra, Luisa Tesoriere

**Affiliations:** 1 Department of Biological, Chemical and Pharmaceutical Sciences and Technologies, University of Palermo, Via Archirafi 28, 90123 Palermo, Italy; ignazio.restivo@unipa.it (I.R.); ileniaconcetta.giardina@unipa.it (I.C.G.); alessandro.massaro01@unipa.it (A.M.); alessandro.attanzio@unipa.it (A.A.); antonella.danneo@unipa.it (A.D.); fanny.pojero@gmail.com (F.P.); luisa.tesoriere@unipa.it (L.T.); 2 Department of Pharmacy, University of Salerno, 84084 Fisciano, Italy; mbasilicata@unisa.it (M.G.B.); esalviati@unisa.it (E.S.); costacolo@unisa.it (C.O.); pcampiglia@unisa.it (P.C.); 3 Department of Biological, Chemical and Pharmaceutical Sciences and Technologies, University of Palermo, Via Archirafi 32, 90123 Palermo, Italy; camilla.pecoraro@unipa.it (C.P.); daniela.carbone@unipa.it (D.C.); stellamaria.cascioferro@unipa.it (S.C.); barbara.parrino@unipa.it (B.P.); patrizia.diana@unipa.it (P.D.)

**Keywords:** polymethoxylated flavones, prenylflavonoids, IBD, inflammation, oxidative stress, phytochemicals

## Abstract

We here investigated the anti-inflammatory activity of a polymethoxylated flavone-containing fraction (PMFF) from *Citrus sinensis* and of a prenylflavonoid-containing one (PFF) from *Humulus lupulus,* either alone or in combination (MIX). To this end, an in vitro model of inflammatory bowel disease (IBD), consisting of differentiated, interleukin (IL)-1β-stimulated Caco-2 cells, was employed. We demonstrated that non-cytotoxic concentrations of either PMFF or PFF or MIX reduced nitric oxide (NO) production while PFF and MIX, but not PMFF, also inhibited prostaglandin E_2_ release. Coherently, MIX suppressed both inducible NO synthase and cyclooxygenase-2 over-expression besides NF-κB activation. Moreover, MIX increased nuclear factor erythroid 2–related factor 2 (Nrf2) activation, heme oxygenase-1 expression, restoring GSH and reactive oxygen and nitrogen species (RONs) levels. Remarkably, these effects with MIX were stronger than those produced by PMFF or PFF alone. Noteworthy, nobiletin (NOB) and xanthohumol (XTM), two of the most represented phytochemicals in PMFF and PFF, respectively, synergistically inhibited RONs production. Overall, our results demonstrate that MIX enhances the anti-inflammatory and anti-oxidative effects of the individual fractions in a model of IBD, via a mechanism involving modulation of NF-κB and Nrf2 signalling. Synergistic interactions between NOB and XTM emerge as a relevant aspect underlying this evidence.

## 1. Introduction

Inflammatory bowel disease (IBD) is a group of relapsing, idiopathic and chronic inflammatory conditions affecting the gastrointestinal tract [1,2]. It is globally prevalent, with increasing incidence in the newly industrialized countries and strictly related to mass consumption of refined sugars and ultra-processed food items, typical of the Westernized diets [3].

While the aetiology of IBD must be considered multifactorial and not clear yet, its development markedly relies on a vicious cycle between oxidative stress and inflammation that eventually impairs the gut barrier structure and function [2,4].

Indeed, alterations of the endocellular redox environment can generate a dysfunctional activation of specific, redox-dependent signal transduction pathways that generates constant and aberrant crosstalk between the immune cells and the intestinal epithelial ones [4,5,6]. These molecular events are orchestrated by an amazingly complex cellular machinery involving the activation of several redox-dependent transcription factors, with nuclear factor erythroid 2-related factor 2 (Nrf2) and nuclear factor kappa-light-chain-enhancer of activated B cells (NF-κB) being key players [7,8]. The first one is responsible for maintaining the endocellular redox balance by activating the antioxidant response element (ARE)-dependent transcription of antioxidant defence enzymes such as heme oxygenase-1 (HO-1), superoxide dismutase-1 (SOD-1) and glutathione S-transferases (GSTs) [9]. On the other hand, NF-κB activation leads to the elaboration of a proinflammatory mediator system, including tumour necrosis factor-α (TNF-α), interleukin (IL)-1β, IL-6, IL-8, prostaglandin E_2_ (PGE_2_) and to the over-expression of crucial, pro-inflammatory enzymes such as inducible nitric oxide synthase (iNOS) and cyclooxygenase 2 (COX-2) [10].

Amongst the pro-inflammatory mediators, IL-1β has repeatedly been reported to play a key role in the intestinal inflammatory response [11]. IL-1β is, indeed, a multifunctional cytokine released by several cell types, including monocytes, macrophages, neutrophils and endothelial cells. Increased levels of IL-1β have been found in the intestinal tissue of IBD patients and linked to higher disease severity [12]. Accordingly, a number of effects of IL-1β on intestinal epithelium have been reported both in vitro and *in vivo*. Indeed, Il-1β activates specific, redox-sensitive, signal transduction pathways that increase the expression of a wide range of pro-inflammatory genes such as those encoding for IL-8, IL-6, TNF-α and iNOS [13,14]. Moreover, a dysfunctional intestinal epithelium can also contribute to the pathogenesis of colitis-related cancer, non-alcoholic fatty liver disease, cardiovascular and kidney diseases and cognitive impairment [15]. All considered, targeting inflammation can be regarded, therefore, as a valid strategy to maintain and improve both intestinal and overall health.

Therapeutical control of IBD is unfortunately limited to a pharmacological approach (corticosteroids and immunomodulators) that is not free from adverse drug reactions [1].

A growing body of evidence strongly suggests that the administration of phytochemicals can support pharmacological therapies, especially for complex and multi-factorial pathological conditions such as inflammation [16]. Indeed, combining anti-inflammatory phytochemicals with standard drugs may reduce drug toxicity and realize a more effective treatment. Moreover, also the combined use of different phytochemicals, targeting different checkpoints of inflammatory diseases, could bring about an enhanced therapeutic outcome by producing synergistic interactions [17].

Over the last decades, citrus (*Citrus sinensis*) fruits and hop (*Humulus lupulus*) have been evaluated for their nutraceutical and health-promoting potential and reported to exert a plethora of beneficial effects in several pathological conditions, including IBD [18,19,20]. The anti-inflammatory effects of citrus and hop strongly depend on their biologically active polyphenols, able to interfere with crucial, redox-dependent signal transduction pathways [21,22].

Flavonoids in citrus juice mainly consist of flavanones and flavones, hesperetin, hesperidin, and naringin are the most abundant flavanones. Among the flavones, tangeretin (TGT) and nobiletin (NOB) are commonly found as polymethoxylated flavones (PMF) in citrus [23,24]. PMF are characterized by multiple methoxy (-OCH3) groups attached to the flavone structure [25]. The number and positions of methoxy groups in PMF significantly influence their bioactivity, with higher methoxy content enhancing hydrophobicity and biological effects [26]. PMF are well-known for their diverse therapeutic properties, including anti-cancer, anti-inflammatory, anti-dysmetabolic, neuroprotective, antimicrobial, and antioxidative activities [26,27,28,29].

Hops are a unique source of natural prenylated flavonoids (PF) [30]. These polyphenols are characterized by the presence of a prenyl group (-C5H8) attached to different positions of the aromatic ring of flavonoids, such as carbon 6 or carbon 8 [31]. The presence of the prenyl group in these compounds plays a significant role in their biological activities enhancing the hydrophobic nature of the molecules and modulating their interactions with target sites such as biomembranes and/or proteins [32]. PF are redox-active molecules able to modulate endocellular redox milieu and thus the redox-dependent signal transduction pathways sustaining the inflammatory response and the dysmetabolic conditions [33,34]. Accordingly, PF exhibits various biological effects including antioxidant, antiviral, antibacterial, antidiabetic, anti-inflammatory, and estrogenic activities [35,36,37,38,39,40,41,42,43].

In the light of the strict interconnections between IBD, oxidative stress and inflammation and considering the anti-oxidative and anti-inflammatory properties of PMF and PF, we here investigated whether these polyphenols subclasses, alone or in combination, display protective effects in an in vitro model of IBD. To this aim, we used selected fractions of PMF (PMFF) and PF (PFF) isolated from the extracts of *Citrus sinensis* and *Humulus lupulus* respectively, either alone or in combination (MIX) as elsewhere reported [44,45]. Moreover, differentiated Caco-2 cells, an established model of the human intestinal barrier, were exposed to IL-1β either in the absence or in the presence of PMFF or PFF or MIX. A number of inflammatory parameters, including pro-inflammatory mediators (NO, PGE_2_), inducible inflammatory enzymes (iNOS and COX-2) and redox-dependent transcription factors (NF-κB and Nrf2) were evaluated. Finally, considering that NOB is one of the major compounds in the PMFF and Xanthohumol (XTM) is one of the main abundant compounds identified in the PFF, we investigated the synergistic effects of NOB and XTM on reactive oxygen and nitrogen species (RONs) levels.

Overall, our results demonstrate for the first time that the association between PMFF and PFF enhances the anti-inflammatory and anti-oxidative effects of the individual fractions in an in vitro model of IBD. The synergistic interactions between NOB and XTM emerge as a relevant aspect underlying this evidence. A mechanistic basis for the observed effects, involving inhibition of NF-κB signalling and stimulation of the Nrf2/HO-1 pathway, is also suggested.

## 2. Materials and Methods

### 2.1. Reagents

Unless otherwise specified, all reagents and chemicals were purchased from Merck (Milan, Italy) and of the highest purity grade available.

### 2.2. PMFF and PFF Isolation

PMFF and PFF were isolated from red-orange fruits and hop flowers, respectively, according to Turdo et al. [35]. Briefly, *Citrus sinensis* fruits var. Tarocco, grown in Salerno, a city located in the Campania region, Italy, was collected in the year 2021, hand-squeezed, and the resulting juice clarified through centrifugation at 15,000× *g* for 15 min at 25 °C and lyophilized. The hop flowers purchased in a local herbalist’s shop were degreased thrice with hexane (1:25, *w*:*v*). The powdered samples of both preparations were subjected to three sequential extractions with methanol (MeOH) for a duration of 10 min each. This extraction process aimed to recover the polyphenolic compounds present in the samples. Finally, PMFF and PFF were purified by reversed-phase semi-preparative liquid chromatography as described by Turdo et al. [35].

### 2.3. Cell Culturing and Treatments

Human, colon adenocarcinoma-derived Caco-2 cells were purchased from Thermo Fisher Scientific (Milan, Italy) and used between passages 15 and 20. Cells were grown in 175 cm^2^ flasks in Dulbecco’s modified Eagle’s medium (DMEM), supplemented with 10% foetal bovine serum (FBS), 1% non-essential amino acids, 10 mM HEPES, 50 units/mL penicillin, 50 mg/mL streptomycin, and maintained at 37 °C in 5% CO_2_. To obtain fully differentiated cells, they were seeded at a density of 1.25 × 10^5^ cells/mL in twenty-four-well plates in DMEM for 21 d. The culture medium was replaced thrice a week. Cell viability was routinely checked by the trypan blue exclusion method.

Before each treatment, differentiated Caco-2 cells were starved overnight in a serum-free medium and then placed in DMEM supplemented with 2% FBS. Differentiated Caco-2 cells were, then, either left untreated (control) or stimulated with IL-1β (25 ng/mL) for either 12, 24 or 48 h. When necessary, cells were preincubated for 1 h with either PMFF or PFF in a concentration range between 2.5 and 20 μg/mL or with a combination of both fractions (1:1, *w*:*w*, MIX) with each fraction at a concentration of 2.5 μg/mL.

### 2.4. Cell Viability

The cytotoxicity of PMFF or PFF against differentiated Caco-2 cells was determined by the MTT colorimetric assay as previously reported [46]. This assay is based on the reduction in 3-(4,5-dimethyl-2-thiazolyl)bromide-2,5-diphenyl-2-H tetrazolium to purple formazan by the mitochondrial dehydrogenases of living cells. Briefly, cells were seeded into 96-well plates (Corning Costar, Milan, Italy) at a density of 2.0 × 10^4^ cells/cm^2^, incubated overnight and then treated either in the absence (control) or in the presence of the fractions for 24 or 48 h. Afterwards, the medium was carefully removed and 200 µL of 5 mg/mL MTT was added. The supernatant was discarded after 2 h of incubation at 37 °C and the formazan blue formed was dissolved in dimethyl sulfoxide. The absorbance at 565 nm that characterise the formazan product was measured using a microplate reader (LTek, INNO, Seongnam, Republic of Korea) and the value of control cells was taken as 100% of viability. Each experiment was repeated three times in triplicate to obtain the mean values.

### 2.5. Nitrite Assay

NO released by differentiated Caco-2 cells in the medium was evaluated as nitrite, by using the Griess reagent as previously reported [47].

### 2.6. Estimation of Combination Index

The Chou and Talalay method [48] was employed to investigate the combined effect of NOB and XTM. Differentiated Caco-2 cells were preincubated for 1 h with either NOB or XTM (in a concentration range between 2.5 and 10 μg/mL) or with MIX, (in a concentration range between 1 and 8 μg/mL). Cells were subsequently treated with IL-1β (25 ng/mL) for 24 h and NO released was determined.

The Combination Index (CI) was calculated using CompuSyn software, 2005 https://www.combosyn.com/register_process.php (accessed on 18 July 2023), (ComboSyn, Paramus, NJ, USA) to define the type of effect. CI is calculated for every fraction affected (*f*_a_) value, where *f*_a_ is defined as percentage inhibition/100. CI > 1, CI < 1 and CI = 1 indicate antagonism, synergism and additive effects, respectively.

### 2.7. PGE_2_ Assay

PGE_2_ released by Caco-2 cells in the medium was measured by a PGE_2_ Enzyme Immunoassay Kit (Cayman Chemical Corporation, Milan, Italy) in accordance with the manufacturer’s instructions. Briefly, after treatment, cells were pelleted by centrifugation at 450× *g* for 5 min at 4 °C. Supernatants were diluted at 1:2.5 with assay buffer. A 100 µL sample, a 50 µL alkaline phosphatase PGE_2_ conjugate and a 50 µL monoclonal anti-PGE_2_ EIA antibody were, then, applied to a goat anti-mouse IgG-containing microtiter plate and incubated at room temperature for 2 h. After washing, 200 µL of p-nitrophenyl phosphate substrate solution was added and incubated at room temperature for 45 min. Finally, optical density at 405 nm was measured on a microplate reader (LTek, INNO, Seongnam, Republic of Korea). PGE_2_ concentrations in the samples were calculated from a PGE_2_ standard curve (25–2000 pg/mL) that was run in parallel.

### 2.8. Western Blot Analysis

Protein levels of COX-2, iNOS and HO-1 were evaluated as previously described [49] with some modifications. Briefly, cells were lysed on ice-cold buffer containing 50 mM Tris-HCl (pH 7.4), 150 mM NaCl, 1 mM EDTA, 1% Triton X-100, 24 mM sodium deoxycholate, 0.01% SDS, 10 mM sodium pyrophosphate, 100 mM sodium fluoride, 10 mM sodium orthovanadate, 1.5 µM aprotinin, 1 mM phenylmethanesulfonylfluoride (PMSF) and 2.1 µM leupeptin. Lysates were centrifuged at 12,000× *g* at 4 °C for 10 min and the supernatants were used for protein determination [50]. Sample buffer (62.5 mM Tris-HCl, 10% glycerol, 2% SDS, 33.2 mM dithiothreitol (DTT) and 0.01% bromophenol blue, pH 6.8) was, then, added to the supernatants. Samples containing 50 µg protein were subjected to SDS-PAGE electrophoresis on 8% acrylamide gels and then electroblotted onto nitrocellulose membranes. Coloured protein markers were used to monitor the progress of protein electrophoresis and to assess the transfer efficiency and the molecular weight of blotted proteins (Amersham^TM^, ECL^TM^ Rainbow^TM^ Marker-Full Range, VWR, Milan, Italy). Membranes were blocked for 2 h in 5% (*w*:*v*) skimmed, dry milk and subsequently incubated in the presence of the corresponding primary antibodies (Santa Cruz, Milan, Italy, 1:1000 dilution) overnight at 4 °C. After incubation for 90 min at room temperature in the presence of secondary, HRP-conjugated antibodies (Dako, Milan, Italy, 1:10,000 dilution), proteins were visualised by using an enhanced chemiluminescent substrate (1.1 mM luminol sodium salt, 2.0 mM 4-iodophenylboronic acid, 5.3 mM hydrogen peroxide and 0.1 M Tris–HCl, pH 8.6). Chemiluminescent bands were evaluated with a C-Digit Blot Scanner (LI-COR, Lincoln, NE, USA) and band intensities were analysed using LI-COR Image Studio 4.0.

To determine the protein levels of either cytosolic or nuclear p65 subunit and Nrf2, corresponding fractions were prepared according to Seubwai et al. [51]. Briefly, cells were lysed in hypotonic buffer (10 mM HEPES KOH at pH 7.9, 1.5 mM MgCl_2_, 10 mM KCl, 1 mM EDTA, 1% NP-40, 0.5 mM DTT, 1 mM PMSF and 10 µg/mL aprotinin). After centrifugation at 2600× *g* for 3 min at 4 °C, the supernatant containing the cytosolic fraction was collected. The pellet was used as the nuclear fraction, lysed with nuclear lysis buffer (20 mM HEPES KOH at pH 7.9, 10% glycerol, 420 mM NaCl, 1.5 mM MgCl_2_, 0.2 mM EDTA, 0.5 mM DTT, 1 mM PMSF and 10 µg/mL aprotinin) and incubated on ice for 30 min. The nuclear fraction was obtained by centrifugation at 21,000× *g* for 10 min at 4 °C. Samples of the nuclear and cytosolic fractions containing 50 µg protein were used for analyses of p65 and Nrf2 levels as above described. All results were expressed as mean ± SD of the densitometric band analysis obtained from three replicates. All results were normalised to β-actin or laminin B. For each protein, a representative lane was selected to compose the figures.

### 2.9. Reactive Oxygen and Nitrogen Species (RONs)

Intracellular levels of RONs were measured by employing a fluorometric detection kit (abcam, Milan, Italy; catalogue number ab113851) according to the manufacturer’s instructions. The assay uses the cell-permeant reagent 2′,7′-dichlorofluorescin diacetate that diffuses through cell membranes and is then deacetylated by cellular esterases to a non-fluorescent compound. This latter is then oxidized by RONs into the highly fluorescent 2′,7′-dichlorofluorescein and detected by fluorescence spectroscopy with excitation/emission at 485 nm/535 nm.

### 2.10. GSH Measurements

Intracellular GSH/GSSG levels were measured by employing a glutathione colorimetric assay kit according to the manufacturer’s instruction (Invitrogen, Milan, Italy, catalogue number EIAGSHC).

### 2.11. Statistical Analysis

Results were reported as mean ± SD. Statistical analysis was performed either by an unpaired Student’s t-test or by ANOVA followed by Tukey’s post hoc test using Prism 8.0, GraphPad (San Diego, CA, USA). Results with a *p*-value < 0.05 were considered statistically significant.

## 3. Results

### 3.1. Evaluation of PMFF and PFF Chemical Composition

In this study, we employed a class-specific isolation approach using reverse-phase semi-preparative liquid chromatography to purify PMFF from citrus juice and PFF from hop flowers.

Fractionation techniques offer several advantages in terms of selectivity, cost-effectiveness, and higher yields over the isolation and purification of individual compounds in the large-scale production of functional ingredients. The isolation of single compounds often involves additional steps that are time-consuming and costly. In contrast, fractionation methods enable the production of larger quantities of bioactive fractions in a shorter time. This approach allows for the selective collection of bioactive compounds based on their structural and chemical similarities. This means that compounds with similar properties and potential benefits can be obtained together, enhancing the overall functionality of the isolated fraction.

**Figure 1:**
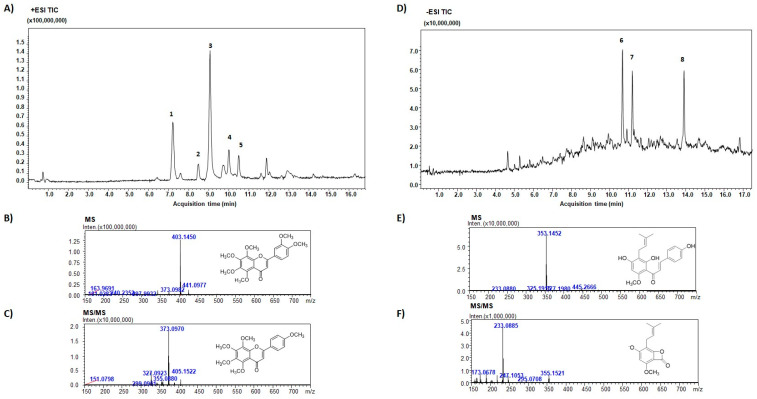
TIC of PMFF isolated from *Citrus sinensis* (**A**); MS (**B**) and MS/MS (**C**) spectra of NOB. #1, SIN; #2, Hexamethoxyflavone; #3, NOB; #4, Heptamethoxyflavone; #5, TGT. TIC of PFF isolated from *Humulus lupulus* (**D**); MS (**E**) and MS/MS (**F**) spectra of XTM. #1, 5,7-Di-O-methyl-8-Prenylnaringenin; #2, Ox-XTM; #3, XTM.

In the present study, we have isolated two aliquots, PMFF or PFF from citrus juice and from hop flowers, respectively. The fractions were collected based on their elution times and thus hydrophobicity. Figure 1 shows the chemical characterization of both PMFF and PFF, including Total ion chromatogram, MS and MS/MS.

LC-MS/MS analysis of PMFF revealed the presence of several biomolecules, including nobiletin (%peak area at 330 nm, 51.0% ± 0.2%), sinensetin (25.3% ± 0.1%), hexamethoxyflavone (3.8% ± 0.1%), heptamethoxyflavone (4.9% ± 0.1%), and tangeretin (5.1% ± 0.3%). MS/MS spectrum showed the characteristic fragment ions of PMFF, displaying a loss of a 31 Da group corresponding to CH_3_O.

On the other hand, the PFF was primarily composed of xanthohumol (% peak area at 370 nm, 27.5% ± 0.2%), ox-xanthohumol (21.8% ± 1.4%), and 5,7-di-O-methyl-8-prenylnaringenin (19.0% ± 1.2%). Figure 1E showed a typical fragmentation pattern of PFF, with the negative charge retained on the A-rings, resulting from retro-Diels-Alder fragmentation.

### 3.2. Evaluation of PMFF and PFF Cytotoxicity, Alone or in Combination, on Differentiated Caco-2 Cells

Before evaluating the anti-inflammatory potential of PMFF and PFF in our experimental system, their cytotoxicity on differentiated Caco-2 cells was assessed by MTT assay. To this aim, cells were either left untreated (control) or incubated with either PMFF or PFF or MIX in a concentration range between 2.5 and 20 µg/mL for 24 h.

**Figure 2:**
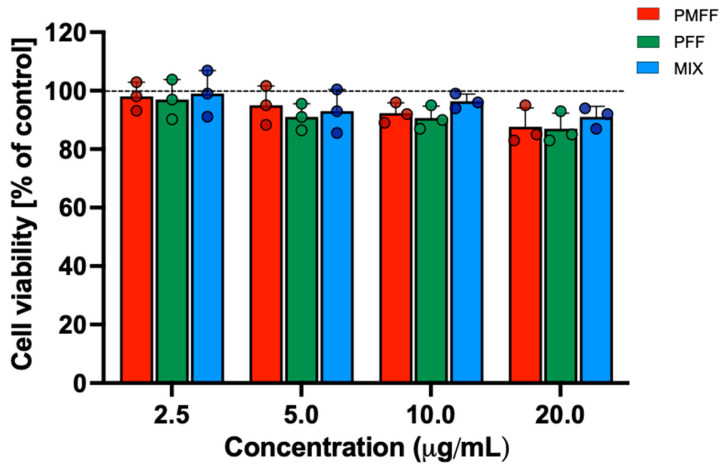
Evaluation of cytotoxicity of PMFF, PFF or MIX on differentiated Caco-2 cells. Cell viability was assessed after a 24 h treatment. Values obtained are the mean ± SD of three separate experiments conducted in triplicates.

As shown in Figure 2, when compared to control cells, neither treatment with PMFF, nor with PFF nor with MIX significantly affected cell viability in the concentration range tested and for the time period evaluated.

### 3.3. PMFF and PFF in Combination Inhibit NO and PGE_2_ Release in Differentiated, IL-1β-Stimulated Caco-2 Cells

NO and PGE_2_ are regarded as two of the most relevant mediators in gastrointestinal inflammatory diseases such as ulcerative colitis and Crohn’s disease [52,53].

The study then continued by assessing the effects of the two fractions on NO and PGE_2_ released by the activated, intestinal cells in our experimental system. To this end, differentiated Caco-2 cells were either left untreated (control) or stimulated with IL-1β (25 ng/mL) for 24 h. When necessary, cells were preincubated with either PMFF (2.5 μg/mL) or PFF (2.5 μg/mL) or MIX (2.5 μg/mL) for 1 h.

**Figure 3:**
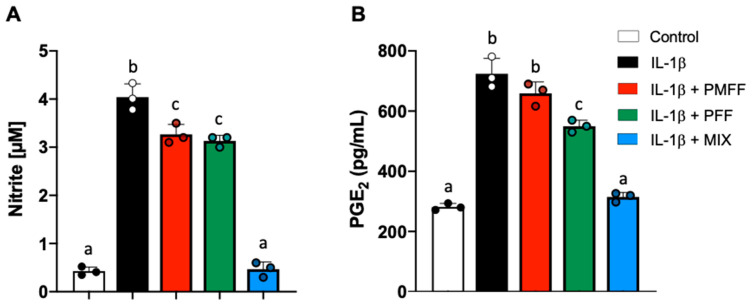
Effect of PMFF, PFF or MIX on NO (**A**) and PGE_2_ (**B**) released from differentiated, IL-1β-activated Caco-2 cells. Values obtained are the mean ± SD of three separate experiments conducted in triplicates. Means with different letters are significantly different with *p* < 0.05 (one-way Anova with Tukey’s post hoc test).

As shown in Figure 3A, with respect to control cells, IL-1β incubation resulted in a significant increase in NO released by Caco-2 cells (*p* < 0.001). Conversely, when Caco-2 cells were pre-incubated for 1 h with either PMFF or PFF at 2.5 μg/mL and then stimulated with IL-1β, a significant (*p* < 0.005) reduction in NO levels was observed with respect to treated cells. Relevantly, pre-incubation with MIX totally prevented IL-1β-induced NO release and brought its value back to control levels (Figure 3A). 

On the other hand, as shown in Figure 3B, a baseline level of PGE_2_ was observed in control cells as a result of the constitutive cyclooxygenase 1 activity. Conversely, IL-1β stimulation significantly (*p* < 0.001) increased PGE_2_ release when compared to control. Interestingly, preincubation of Caco-2 cells with PMFF did not significantly affect PGE_2_ production with respect to IL-1β-treated cells. On the other hand, a significant (*p* < 0.02) reduction was observed in the presence of PFF vs. treated cells. Relevantly, pre-incubation with MIX totally prevented IL-1β-induced PGE_2_ release and brought its value back to.

### 3.4. PMFF and PFF in Combination Reduce iNOS and COX-2 Expression Levels in Differentiated, IL-1β-Activated Caco-2 Cells

Since MIX treatment strongly decreased NO and PGE_2_ release from differentiated IL-1β-activated Caco-2 cells, we then investigated whether PMFF or PFF treatment (either individually or in combination) affected iNOS and COX-2 protein levels. To this aim, differentiated Caco-2 cells were either left untreated (control) or stimulated with IL-1β (25 ng/mL) for 24 h. When necessary, cells were preincubated with either PMFF (2.5 μg/mL) or PFF (2.5 μg/mL) or MIX (2.5 μg/mL) for 1 h.

**Figure 4:**
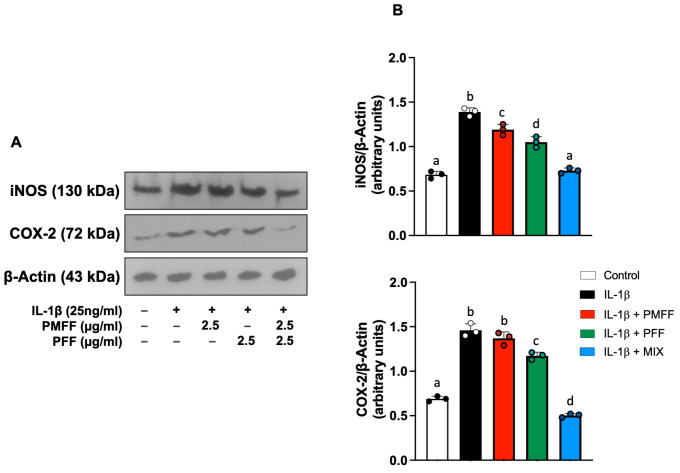
Effect of PMFF, PFF or MIX on iNOS and COX-2 protein levels in differentiated, IL-1β activated Caco-2 cells. Representative images of Western blot analysis (**A**). Densitometric analysis of iNOS and COX-2 protein levels normalised for β actin levels (**B**). Values obtained are the mean ± SD of three separate experiments. Means with different letters are significantly different with *p* < 0.05 (one-way Anova with Tukey’s post hoc test).

As shown in Figure 4, stimulation of cell monolayers with Il-1β induced a significant (*p* < 0.001) increase in both iNOS and COX-2 protein levels as compared to control. On the other hand, and coherently with the above-reported results on NO levels, co-incubation of cells with either PMFF or PFF significantly reduced the IL-1β-induced upregulation of iNOS (*p* < 0.005 and *p* < 0.001 respectively). Moreover, and in line with the observed effects on PGE_2_ release, PMFF did not induce any significant inhibition on COX-2 overexpression, while PFF treatment significantly reduced COX-2 protein levels (*p* < 0.001). Relevantly, co-incubation with MIX reduced iNOS and COX-2 overexpression either to control levels or below, respectively.

### 3.5. PMFF and PFF in Combination Potentiate the Inhibition of IL-1β-Dependent Activation of NF-κB in Differentiated Caco-2 Cells

Our investigation next evaluated whether the reduction in IL-1β-induced COX-2 and iNOS over-expression by PMFF and PFF was associated with an inhibition of NF-κB activation. To this aim, differentiated Caco-2 cells were either left untreated (control) or stimulated with IL-1β (25 ng/mL) for 12 h. When necessary, cells were preincubated with either PMFF (2.5 μg/mL) or PFF (2.5 μg/mL) or MIX (2.5 μg/mL) for 1 h. The translocation of the p65 subunit to the nucleus was assessed as a marker of NF-κB activation.

**Figure 5:**
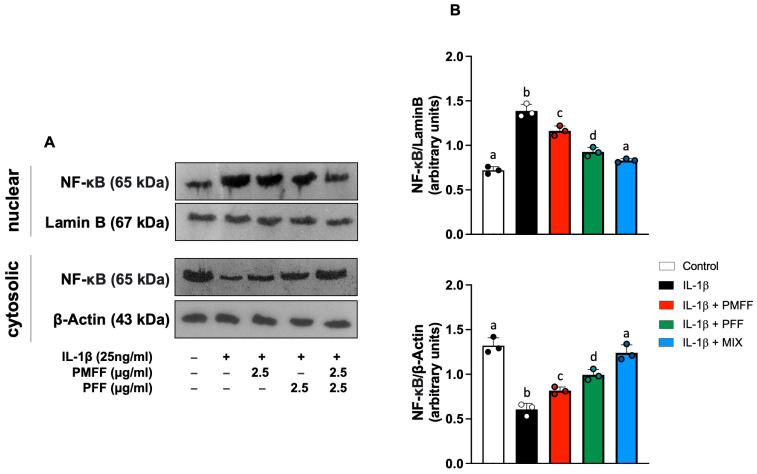
Effect of PMFF, PFF or MIX on p65 nuclear translocation in differentiated, IL-1β-activated Caco-2 cells. Representative images of Western blot analysis (**A**). Densitometric analysis of p65 protein levels normalised for β actin or laminin b levels (**B**). Values obtained are the mean ± SD of three separate experiments. Means with different letters are significantly different with *p* < 0.05 (one-way Anova with Tukey’s post hoc test).

As shown in Figure 5, when compared to control, stimulation with IL-1β resulted in a significant increase in NF-κB nuclear translocation, evident from the increased nuclear levels of p65 and the concomitant reduction in the cytosolic ones (*p* < 0.001). On the other hand, PMFF or PFF pre-treatment induced a significant reduction in p65 nuclear levels (*p* < 0.005 and *p* < 0.001, respectively). Interestingly, MIX preincubation completely inhibited IL-1β-induced p65 nuclear translocation, restoring nuclear and cytosolic protein levels to control ones.

### 3.6. PMFF and PFF in Combination Enhances the Nuclear Translocation of Nrf2 and the Expression of HO-1 in Differentiated Caco-2 Cells

Taking into account the present results on the inhibition by PMFF and PFF of both NF-κB activation and the expression of its downstream proinflammatory products, we next investigated whether the two fractions, either alone or in combination, would also affect Nrf2 signalling. To this aim, differentiated Caco-2 cells were either left untreated (control) or stimulated with IL-1β (25 ng/mL) for 12 h. When necessary, cells were preincubated with either PMFF (2.5 μg/mL) or PFF (2.5 μg/mL) or MIX (2.5 μg/mL) for 1 h. The nuclear translocation of Nrf2 was assessed as a marker of its activation.

**Figure 6:**
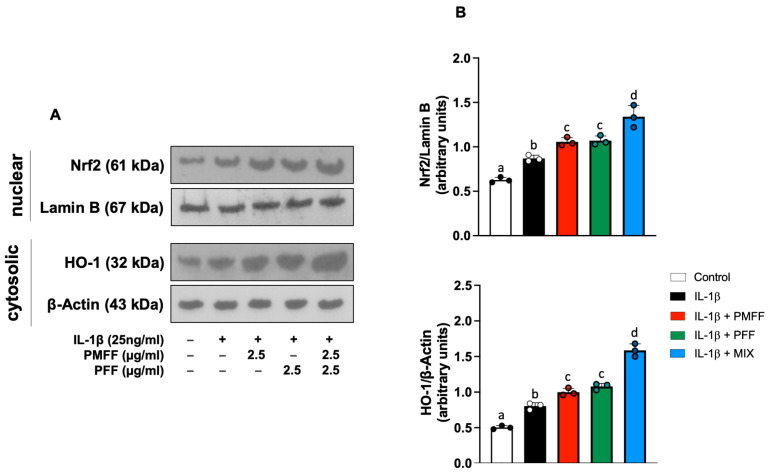
Effect of PMFF, PFF or MIX on either Nrf2 nuclear translocation or cytosolic levels of HO-1 in differentiated, IL-1β-activated Caco-2 cells. Representative images of Western blot analysis (**A**). Densitometric analysis of either Nrf2 or HO-1 protein levels normalised for laminin b or β actin levels, respectively (**B**). Values obtained are the mean ± SD of three separate experiments. Means with different letters are significantly different with *p* < 0.05 (one-way Anova with Tukey’s post hoc test).

As shown in Figure 6, when compared to control cells, stimulation with IL-1β increases Nrf2 nuclear translocation (*p* < 0.01). Interestingly, both PMFF and PFF individually added, significantly increased Nrf2 activation (*p* < 0.05) with respect to IL-1β-treated cells. Remarkably, a significant further increase in its nuclear levels was evident when PMFF and PFF were added in combination (*p* < 0.001).

To further characterise the antioxidative response of the cells, induced by the fractions, the effect of PMFF and PFF on the protein expression of HO-1, an antioxidant enzyme representative of the transcriptional activity of Nrf2, was then evaluated. To this end, Caco-2 cells were treated as above described and stimulated with Il-1β for 24 h. As shown in Figure 6, with respect to control cells, stimulation with IL-1β increases HO-1 protein levels (*p* < 0.001). Moreover, preincubation with either PMFF or PFF induced a significant HO-1 protein over-expression with respect to IL-1β-treated cells (*p* < 0.01). Relevantly, MIX treatment generated a significantly higher increase in HO-1 protein levels with respect to cells incubated in the presence of the individual fractions (*p* < 0.001).

### 3.7. PMFF and PFF in Combination Enhances the Antioxidative Response in Differentiated, IL-1β-Activated Caco-2 Cells

**Figure 7:**
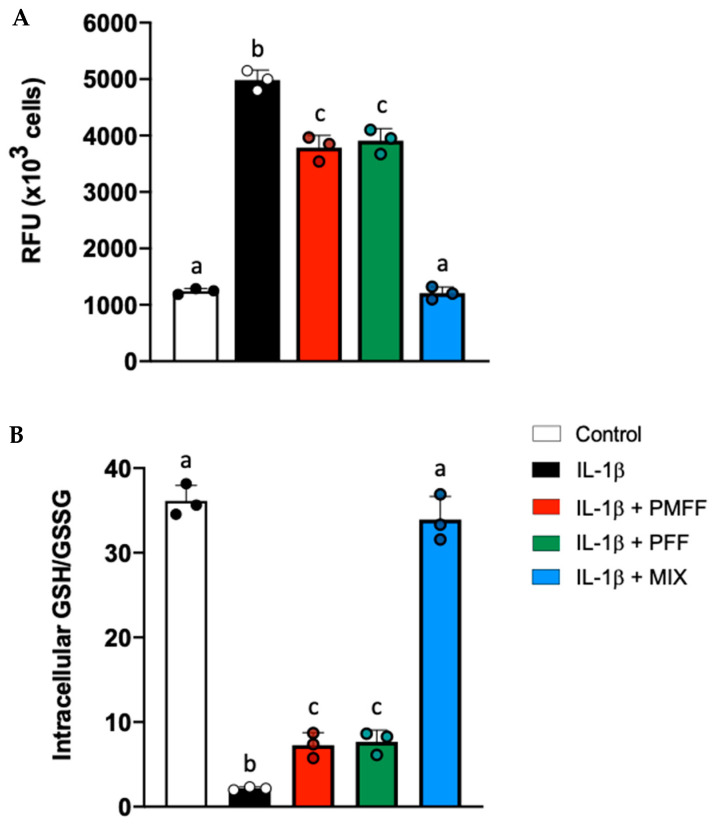
Effect of PMFF, PFF or MIX on IL-1β-induced cellular redox unbalance in activated, differentiated Caco-2 cells. RONs, expressed as relative fluorescence units (RFU), (**A**) and GSH/GSSG (**B**). Values obtained are the mean ± SD of three separate experiments in triplicate. Means with different letters are significantly different with *p* < 0.05 (one-way Anova with Tukey’s post hoc test).

By regulating the expression of key antioxidative enzymes, Nrf2 is a key modulator of cellular redox balance [54]. Finally, we measured both endocellular RONs and GSH levels in our experimental system. To this aim, differentiated Caco-2 cells were either left untreated (control) or stimulated with IL-1β (25 ng/mL) for 24 h. When necessary, cells were preincubated with either PMFF (2.5 μg/mL) or PFF (2.5 μg/mL) or MIX (2.5 μg/mL) for 1 h. As shown in Figure 7, with respect to control cells, treatment with IL-1β caused a significant increase in RONs levels and a decrease in the GSH/GSSG ratio, indicating a redox unbalance of cells towards a more oxidized state (*p* < 0.001). Interestingly, pre-treatment with either PMFF or PFF (2.5 μg/mL) significantly counteracted the IL-1β-induced RONs production and the reduction in GSH/GSSG ratio (*p* < 0.001 and *p* < 0.05, respectively). Remarkably, MIX pre-treatment totally prevented the IL-1β-induced redox unbalance, by restoring RONs levels and GSH/GSSG ratio back to control values.

### 3.8. NOB and XTM in Combination Synergistically Counteract Endocellular Redox Unbalance in Differentiated, IL-1β-Activated Caco-2 Cells

As NOB and XTM were identified as the most abundant compounds of the PMFF and the PFF, respectively, we finally investigated whether the redox-modulating effects exerted by the two fractions were associated with the ability of these phytochemicals to synergistically reduce the IL-1β-induced RONs production in our experimental system.

**Figure 8:**
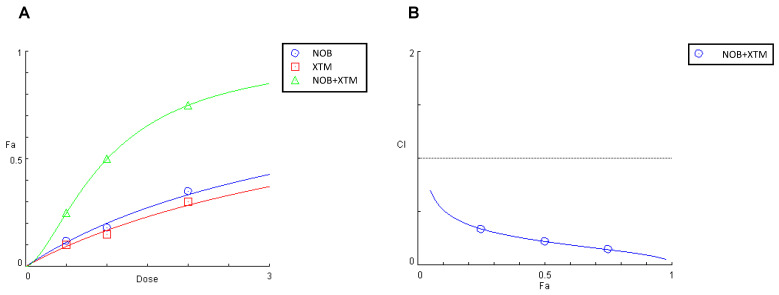
Effect on RONs release in IL-1β-stimulated Caco-2 cells of NOB (0.5–2.0 μM), XTM (0.5–2.0 μM), and NOB combined with XTM (molar ratio of 1:1) (**A**) and plot of the combination index vs. fraction of the inhibitory effect (**B**). Cells were pre-treated individually or in combination with NOB and XTM for 1 h. The interactions on inhibitory effects were analysed using the median-effect analysis program where CI > 1, CI < 1 and CI =1 indicate an antagonistic, synergistic or additive effect, respectively.

To this aim, we followed the Chou–Talalay Combination Index (CI) model, based on the median effect equation. The CI values for the different inhibition fractions (fa) of RONs production and represented the data as a function of the measured effect (Figure 8B). According to Chou–Talalay, values of CI > 1, CI < 1 and CI = 1 indicate an antagonistic, synergistic or additive effect, respectively. In our study the CI values measured for the release of RONs by IL-1β-stimulated Caco-2 cells, were all far below 1, indicating a synergistic inhibitory effect of NOB and XTM in a wide range of concentrations.

## 4. Discussion

This work falls within the intense research on the advantage of combining different phytochemicals to improve the health benefits of single compounds. While the anti-inflammatory effects of PMFF and PFF are well established and both classes of compounds have been shown to exert significant protective effects in several in vitro and in vivo models of intestinal diseases, no study has yet evaluated their efficacy when added in combination with such experimental systems. Interestingly, we here demonstrate that a combination of PMFF and PFF exerts more effective anti-inflammatory effects in IL-1β-activated, intestinal cells with respect to a treatment with either PMFF or PFF alone. Inhibition of the redox-dependent NF-κB/Nrf2 activation and of their downstream signalling axis appears as key mechanisms underlying the PMFF- and PFF-mediated anti-inflammatory effects.

NO is a pleiotropic free radical messenger molecule, responsible for several physiological functions of the gastrointestinal mucosa such as the maintenance of perfusion, the regulation of microvascular epithelial permeability and the modulation of the immune response [52]. Increased output production of NO by intestinal cells, however, has been correlated to intestinal injury, mucosal inflammation, and enterocyte apoptosis through mechanisms correlated to the increased nitrosative stress and DNA damage [52,55,56]. Along these lines, the ability of PMFF and PFF to inhibit NO release in our in vitro model of IBD appears remarkable. Moreover, our data are in agreement with published evidence demonstrating the effectiveness of NOB and XTM to reduce NO production in an in vivo model of colitis, and the efficacy of SIN and TGT in a number of in vitro models of immunological dysfunctions in several cell types [19,57,58,59,60,61].

PGE_2_ is one of the most relevant downstream products of COX-2 enzymatic activity. Together with NO, it modulates several physiological intestinal functions such as stimulation of mucus, bicarbonate secretion, mucosal blood flow, leucocyte recruitment, fever and pain [62,63]. At the same time, multiple lines of evidence demonstrated that PGE_2_ is a key mediator of IBD, exerting significant pro-inflammatory and tumor-promoting effects [53]. Indeed, PGE_2_ overproduction by intestinal cells has been reported to exacerbate the inflammatory process, inducing the disruption of the intestinal epithelial barrier function and promoting proinflammatory Th17 cell signalling [64,65]. Interestingly, our results demonstrated that PFF, but not PMFF, is able to significantly reduce the release of PGE_2_ in our experimental system. Relevantly, when PMFF acts in combination with PFF the phytochemicals exert a much more evident anti-inflammatory response, reducing PGE_2_ levels to control. This evidence further reinforces the concept that the association between the two fractions can be envisaged as a rational and effective anti-inflammatory strategy for the treatment of IBD.

The present effects of PMFF and PFF on NO and PGE_2_ levels mirror their effects on both iNOS and COX-2 expression, observed in our experimental system. Indeed, and coherently with the ability of both fractions to inhibit the release of NO, either PMFF or PFF counteract IL-1β-induced iNOS over-expression. Conversely, and in line with the incapacity of PMFF to inhibit PGE_2_ release, this fraction is not able to reduce COX-2 levels. Remarkably, only the contemporaneous presence of both PMFF and PFF results in the inhibition of both enzymes, reducing iNOS or COX-2 expression to control levels or even below, respectively. These effects, obtained only when the fractions were combined, appear of particular interest as both these pro-inflammatory enzymes are dramatically upregulated in IBD patients. Indeed, intestinal chronic inflammation can orchestrate the development of a tumour-supporting microenvironment that promotes tumour initiation, progression and metastasis. Remarkably, COX-2 over-expression plays a key role in IBD-associated colon rectal cancer (CRC) being strongly associated with worse survival among CRC patients [66,67]. Therefore, the simultaneous inhibition of iNOS and COX-2 is crucial to achieve maximal protection as demonstrated in chemically-induced colitis [68], colitis-related carcinogenesis and CRC development in patients with IBD [69,70,71]. Within this scenario, these results relevantly underlie how important is the combination of the PMFF and PFF, which appears necessary to achieve the contemporary inhibition of both iNOS and COX-2.

The IL-1β-induced rapid transcription of *iNOS*, *COX-2* and several other crucial genes involved in the inflammatory response, strongly involves the activation of the redox-sensitive transcription factor NF-κB [72]. A growing number of evidence has revealed how critical is the role of NF-κB activation on both the onset and the progression of IBD [73,74]. Upon activation, the protein translocates from the cytoplasm to the nucleus, where binds to specific DNA sequences, initiating the transcription of its downstream target genes [75]. Interestingly, our results indicate that both PMFF and PFF are able to inhibit NF-κB activation and, more importantly, that only when are added in combination do they bring back the nuclear levels of the transcription factor to control values. These effects can be related to previously published evidence showing that XTM inhibits NF-κB nuclear translocation by counteracting IκBα phosphorylation both in vitro and in vivo [20] and to the ability of NOB, SIN and TGT to inhibit the NF-κB pathway [18,76,77,78,79,80].

Nrf2 is a key transcription factor controlling many aspects of cell homoeostasis in response to oxidative and toxic insults. Indeed, Nrf2 modulates the transcription of several cytoprotective, detoxification and antioxidant genes such as HO-1 [54,81]. NOB, SIN, TGT and XTM have been previously demonstrated to positively modulate Nrf2 activation and HO-1 protein levels in other experimental systems [82,83,84,85,86,87,88,89]. Accordingly, our results demonstrate that either PMFF or PFF can further increase the IL-1β-induced Nrf2 activation and HO-1 over-expression in an in vitro model of IBD. Remarkably, present data also show, for the first time, that the combination of both fractions results in an enhanced antioxidative response (in terms of Nrf2 activation and HO-1 overexpression) with respect to the one induced by the treatment with the single fractions, in our experimental system.

Moreover, a growing number of evidence demonstrates that the Nrf2 function is strictly integrated and interconnected with NF-κB signalling [90]. Indeed, Nrf2 can negatively regulate the NF-κB activation pathway, counteracting NF-κB-related pro-inflammatory response and oxidative injury [91,92]. Along these lines, it is tempting to speculate that the inhibition of NF-κB observed in this work may be related to the activation of Nrf2 and the subsequent overexpression of HO-1 by PMFF and PFF treatment.

GSH is a ubiquitous intracellular peptide with diverse functions that helps to scavenge RONs, reduces peroxides and reacts with electrophilic compounds providing cells with multiple defences against RONs and their by-products [93]. Consistent with the activation of the Nrf2/HO-1 axis by PMFF and PFF, our results also showed for the first time, in an in vitro model of IBD, a significant amelioration of the endocellular oxidative stress, in terms of RONs reduction and GSH levels increase. These results are in line with the antioxidative effects exerted by the phytochemicals of both fractions in other IBD-unrelated experimental models either in vitro or in vivo [82,84,86,87,88,89,94]. Relevantly, these effects were significantly more evident when both fractions were combined and might well be related to the Nrf2-dependent enhancement of the endogenous antioxidant defence systems by the phytochemicals, especially when present in combination.

Finally, our current results relevantly demonstrate for the first time the ability of NOB and XTM, the most abundant components of PMFF and PFF, respectively, to synergistically reduce the IL-1β-induced RONs production in differentiated and activated Caco-2 cells. While suggesting that the redox-modulating effects exerted by the two fractions could be associated with the synergistic interactions between these phytochemicals, this evidence could also foster further research aimed to explore other relevant synergistic interactions between the phytochemicals of PMFF and PFF.

## 5. Conclusions

As a whole, our data support the concept that phytocomplexes can potentiate the health effects of their single components. Indeed, we here demonstrate for the first time that PMFF and PFF exert more effective anti-inflammatory effects in an in vitro model of IBD when added in combination. Modulation of the redox-dependent NF-κB/Nrf2 activation and of their downstream signalling axis appears key mechanisms underlying the PMFF- and PFF-mediated anti-inflammatory effects. Synergistic interactions between NOB and XTM emerge as a relevant aspect underlying this evidence.

## Data Availability

Data supporting reported results can be requested to the corresponding authors.
